# Pathobiology of the Klotho Antiaging Protein and Therapeutic Considerations

**DOI:** 10.3389/fragi.2022.931331

**Published:** 2022-07-12

**Authors:** Gérald J. Prud’homme, Mervé Kurt, Qinghua Wang

**Affiliations:** ^1^ Department of Laboratory Medicine and Pathobiology, University of Toronto, Toronto, ON, Canada; ^2^ Department of Laboratory Medicine, Keenan Research Centre for Biomedical Science, Unity Health Toronto, Toronto, ON, Canada; ^3^ Department of Endocrinology and Metabolism, Huashan Hospital, Shanghai Medical School, Fudan University, Shanghai, China; ^4^ Shanghai Yinuo Pharmaceutical Co., Ltd., Shanghai, China

**Keywords:** aging, FGF23, hyperphosphatemia, klotho, IGF-1, NF-KappaB, TGF-beta, Wnt

## Abstract

The α-Klotho protein (henceforth denoted Klotho) has antiaging properties, as first observed in mice homozygous for a hypomorphic *Klotho* gene (*kl/kl*). These mice have a shortened lifespan, stunted growth, renal disease, hyperphosphatemia, hypercalcemia, vascular calcification, cardiac hypertrophy, hypertension, pulmonary disease, cognitive impairment, multi-organ atrophy and fibrosis. Overexpression of Klotho has opposite effects, extending lifespan. In humans, Klotho levels decline with age, chronic kidney disease, diabetes, Alzheimer’s disease and other conditions. Low Klotho levels correlate with an increase in the death rate from all causes. Klotho acts either as an obligate coreceptor for fibroblast growth factor 23 (FGF23), or as a soluble pleiotropic endocrine hormone (s-Klotho). It is mainly produced in the kidneys, but also in the brain, pancreas and other tissues. On renal tubular-cell membranes, it associates with FGF receptors to bind FGF23. Produced in bones, FGF23 regulates renal excretion of phosphate (phosphaturic effect) and vitamin D metabolism. Lack of Klotho or FGF23 results in hyperphosphatemia and hypervitaminosis D. With age, human renal function often deteriorates, lowering Klotho levels. This appears to promote age-related pathology. Remarkably, Klotho inhibits four pathways that have been linked to aging in various ways: Transforming growth factor β (TGF-β), insulin-like growth factor 1 (IGF-1), Wnt and NF-κB. These can induce cellular senescence, apoptosis, inflammation, immune dysfunction, fibrosis and neoplasia. Furthermore, Klotho increases cell-protective antioxidant enzymes through Nrf2 and FoxO. In accord, preclinical Klotho therapy ameliorated renal, cardiovascular, diabetes-related and neurodegenerative diseases, as well as cancer. s-Klotho protein injection was effective, but requires further investigation. Several drugs enhance circulating Klotho levels, and some cross the blood-brain barrier to potentially act in the brain. In clinical trials, increased Klotho was noted with renin-angiotensin system inhibitors (losartan, valsartan), a statin (fluvastatin), mTOR inhibitors (rapamycin, everolimus), vitamin D and pentoxifylline. In preclinical work, antidiabetic drugs (metformin, GLP-1-based, GABA, PPAR-γ agonists) also enhanced Klotho. Several traditional medicines and/or nutraceuticals increased Klotho in rodents, including astaxanthin, curcumin, ginseng, ligustilide and resveratrol. Notably, exercise and sport activity increased Klotho. This review addresses molecular, physiological and therapeutic aspects of Klotho.

## 1 Introduction

Aging is associated with changes in almost all tissues and organs of the body, resulting eventually in debilitating and/or fatal conditions such as cardiovascular disease, chronic organ failure, neurodegeneration and cancer. Aging occurs at varying speed in different species, suggesting some poorly defined biological clock. The molecular mechanisms underlying aging are intensely studied, but still not well understood. However, the pathogenesis of age-associated diseases is likely multi-factorial (Franceschi et al., 2018). In humans, a limited number of genes have been clearly linked to accelerated aging as seen in progeria (a rare condition) (Coppede 2021) or, conversely, associated with longevity (Javidnia et al., 2022). The subject of this review is *Klotho (kl)*, which is an antiaging gene, and the corresponding protein α-Klotho (henceforth denoted Klotho or KL). The gene was first identified in mice in 1997 (Kuro-o et al., 1997). Deficiency of the protein results in a syndrome that has several features of aging, as observed in mutant mice with either a hypomorphic *Klotho* allele (*Kl* ^*kl/kl*^) (Kuro-o, et al., 1997) or full knockout of the *Klotho* gene (*Kl* ^**−/−**^) (Tsujikawa et al., 2003). Klotho-deficient mice exhibit stunted growth, renal disease, hyperphosphatemia, hypercalcemia, vascular calcification, cardiac hypertrophy, hypertension, organ fibrosis, multi-organ atrophy, osteopenia, pulmonary disease, cognitive impairment and short lifespan (Bian et al., 2015; Erben and Andrukhova, 2017; Kinoshita and Kawai, 2016; Kuro-o et al., 1997, 2010, 2019, 2021). Overexpression of the gene has the opposite effects, lengthening survival (Kurosu et al., 2005).

Klotho insufficiency appears to play a role in human aging and, specifically, in many of the diseases that are associated with aging. Klotho expression declines with age, renal failure, diabetes and neurodegenerative disease. The age-related decline in serum levels appears to be similar in men and women; and reference values have recently been reported (Espuch-Oliver et al., 2022). Notably, a recent study of American adults showed that low serum Klotho levels correlate with an increased all-cause death rate (Kresovich and Bulka, 2021).

**FIGURE 1:**
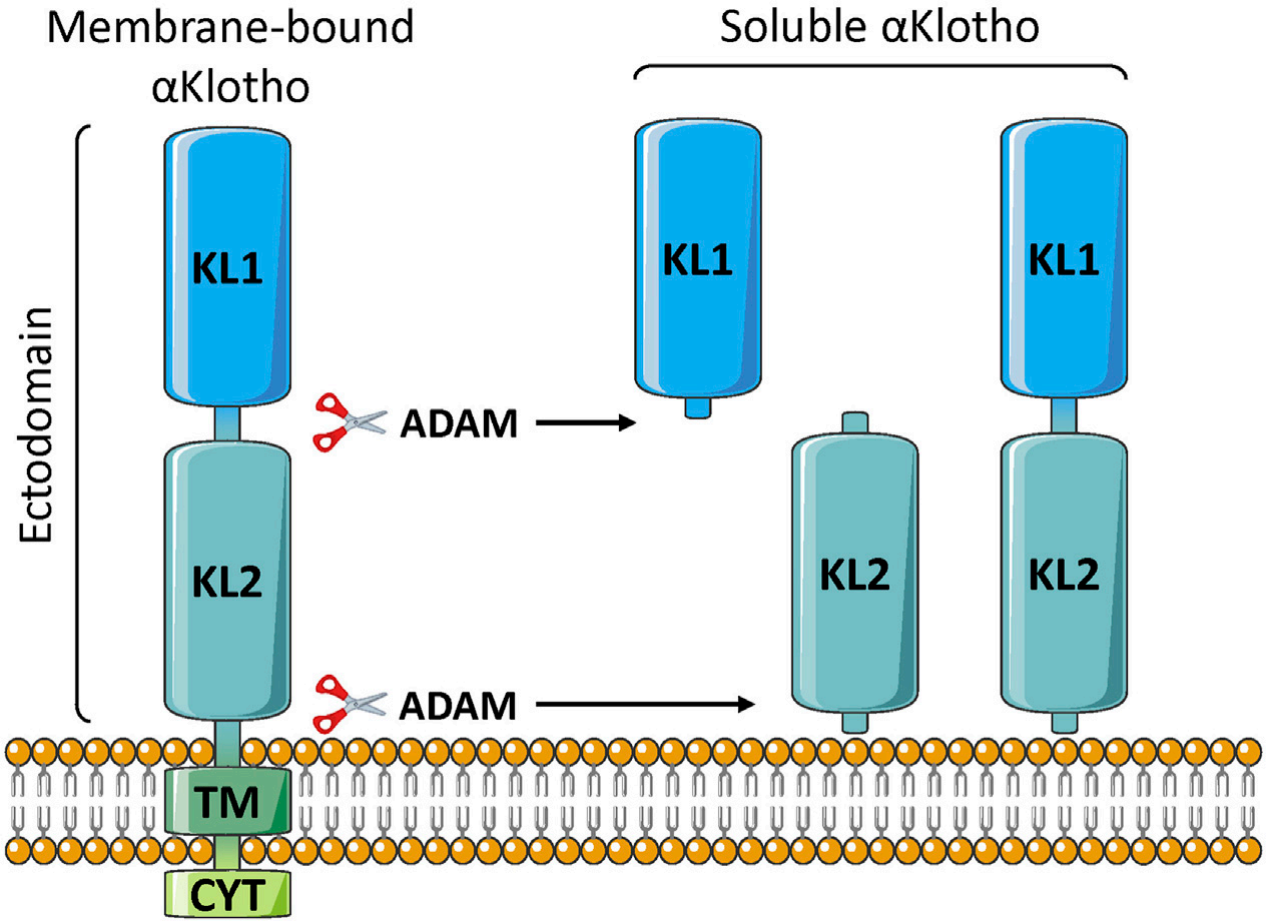
Klotho structure. The membrane-bound form is single-pass, and consists of two extracellular domains (KL1 and KL2), a transmembrane segment (TM), and a short non-signaling cytoplasmic tail (CYT). The soluble form is generated by proteolytic cleavage, usually by either ADAM10 or ADAM17 enzymes, to release the large soluble form (s-Klotho). This is the major form found in the circulation. It can be further cleaved to generate independent KL1 and KL2 fragments, but these are either minor forms or undetectable in the plasma.

**FIGURE 2:**
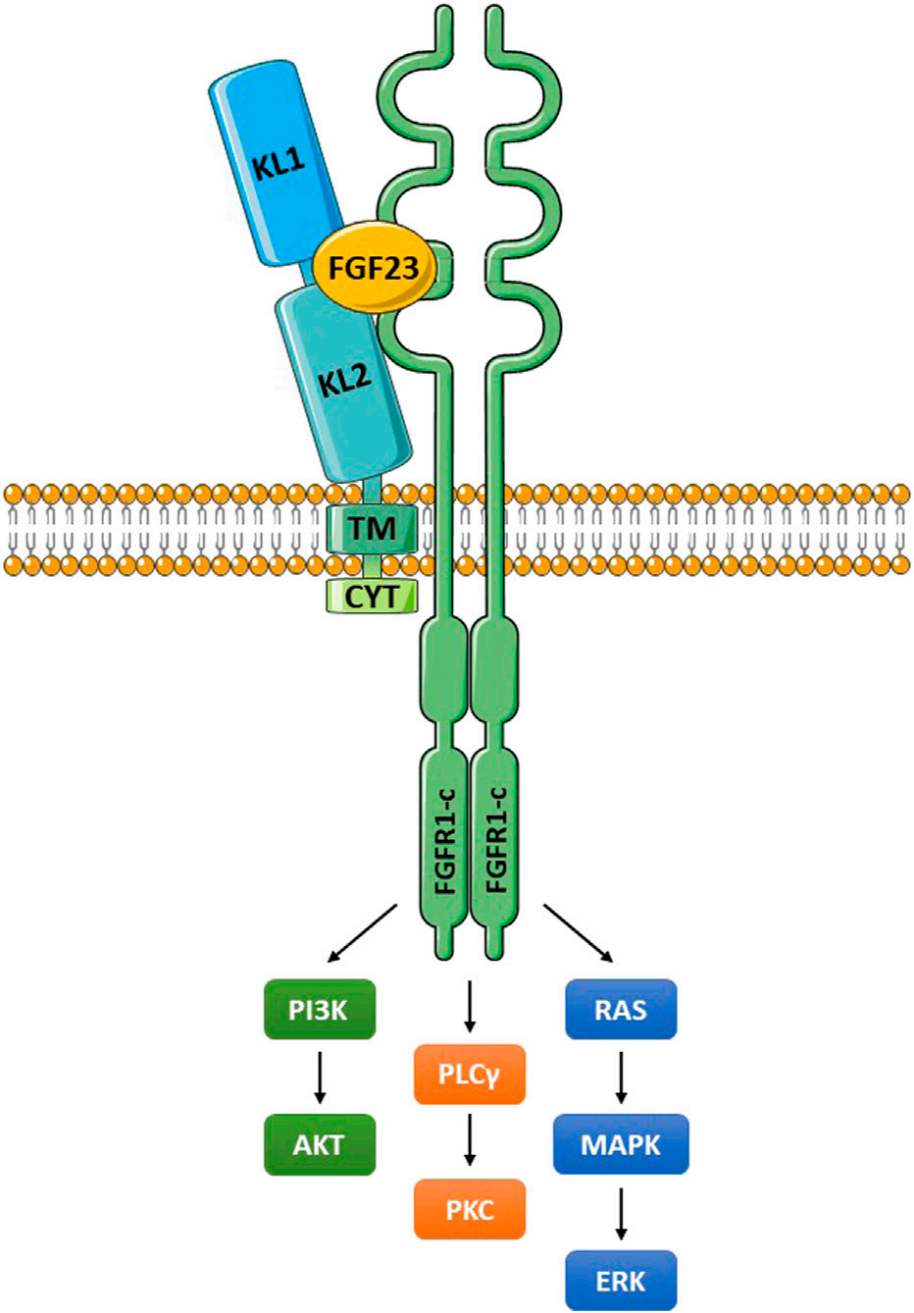
The Klotho/FGFR/FGF23 signaling complex. Klotho interacts with a FGFR (frequently FGFR1c) through an extension of its KL2 domain. FGF23 fits into a groove formed by components of KL1, KL2 and the FGFR. The membrane-bound and soluble Klotho forms (KL1/KL2 domains) can both bind to FGFR1c and function as coreceptors (Chen G, et al., 2018). These molecular interactions create a high affinity binding site for FGF23. The activated FGFR signals through multiple pathways as illustrated, and outlined in the text. Abbreviations: ERK, extracellular signal-regulated kinase; FGF23, fibroblast growth factor 23; FGFR1-c, FGF receptor 1c; MAPK, mitogen-activated protein kinase; PI3K, phosphoinositide 3-kinase; PKC, protein kinase C; PLCγ, phospholipase Cγ.

**FIGURE 3:**
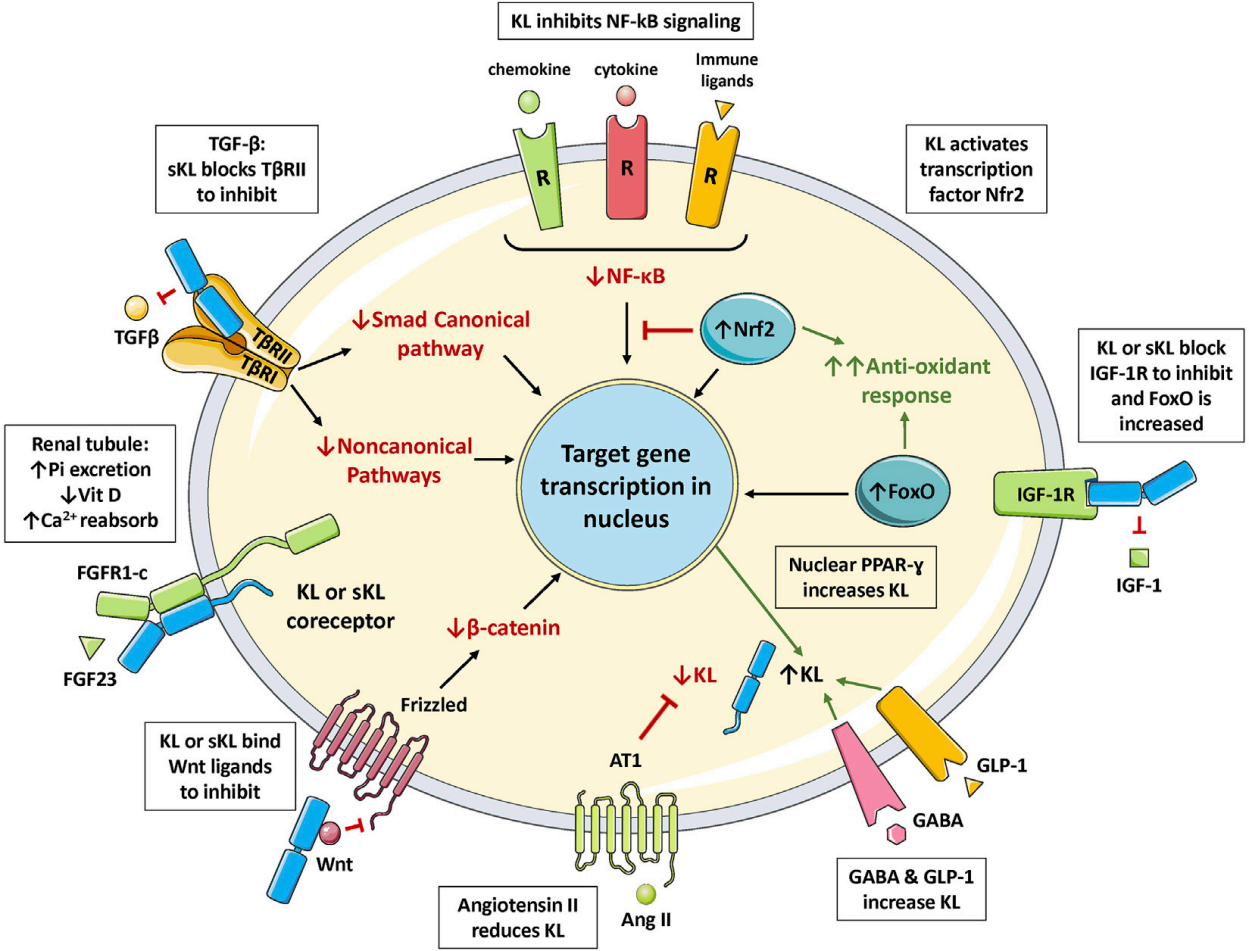
The multiple functions of Klotho. FGF23 binds to Klotho/FGFR1c receptor in the renal tubule to increase excretion of phosphate (phosphaturic effect), regulate vitamin D metabolism, and increase calcium reabsorption. Klotho binds to the TGF-β receptor (TβRII component) to block TGF-β action. Klotho inhibits activation of the inflammatory NF-κB pathway by preventing nuclear translocation of the active form. Klotho increases signaling in the Nrf2 pathway; inducing multiple antioxidant enzymes and inhibiting NF-κB. Klotho blocks IGF-1 receptor signaling; this increases activation of FoxO and antioxidant responses. Klotho also blocks activation of the Wnt pathway by binding to soluble Wnt ligands. Klotho expression is increased by PPAR-γ activation, and also by GLP-1 and GABA stimulation; but is suppressed by angiotensin II activation of the AT1 receptor. Abbreviations: Ang II, angiotensin II; Ca2+, calcium ion; FoxO, forkhead boxprotein O; GABA, γ-aminobutyric acid; GLP-1, glucagon-like peptide 1; IGF-1, insulin-like growth factor 1; IGFR, IGF-1 receptor; KL, membrane-bound αKlotho; NF-κB, nuclear factor κB; Nrf2, nuclear factor-erythroid 2-related factor 2; Pi, inorganic phosphate; PPAR-γ, peroxisome proliferator-activated receptors γ; R, receptor; sKL, soluble αKlotho; TGF-β, transforming growth factor β; TβRII, TGF-β receptor type II; Vit D, vitamin D.

**FIGURE 4:**
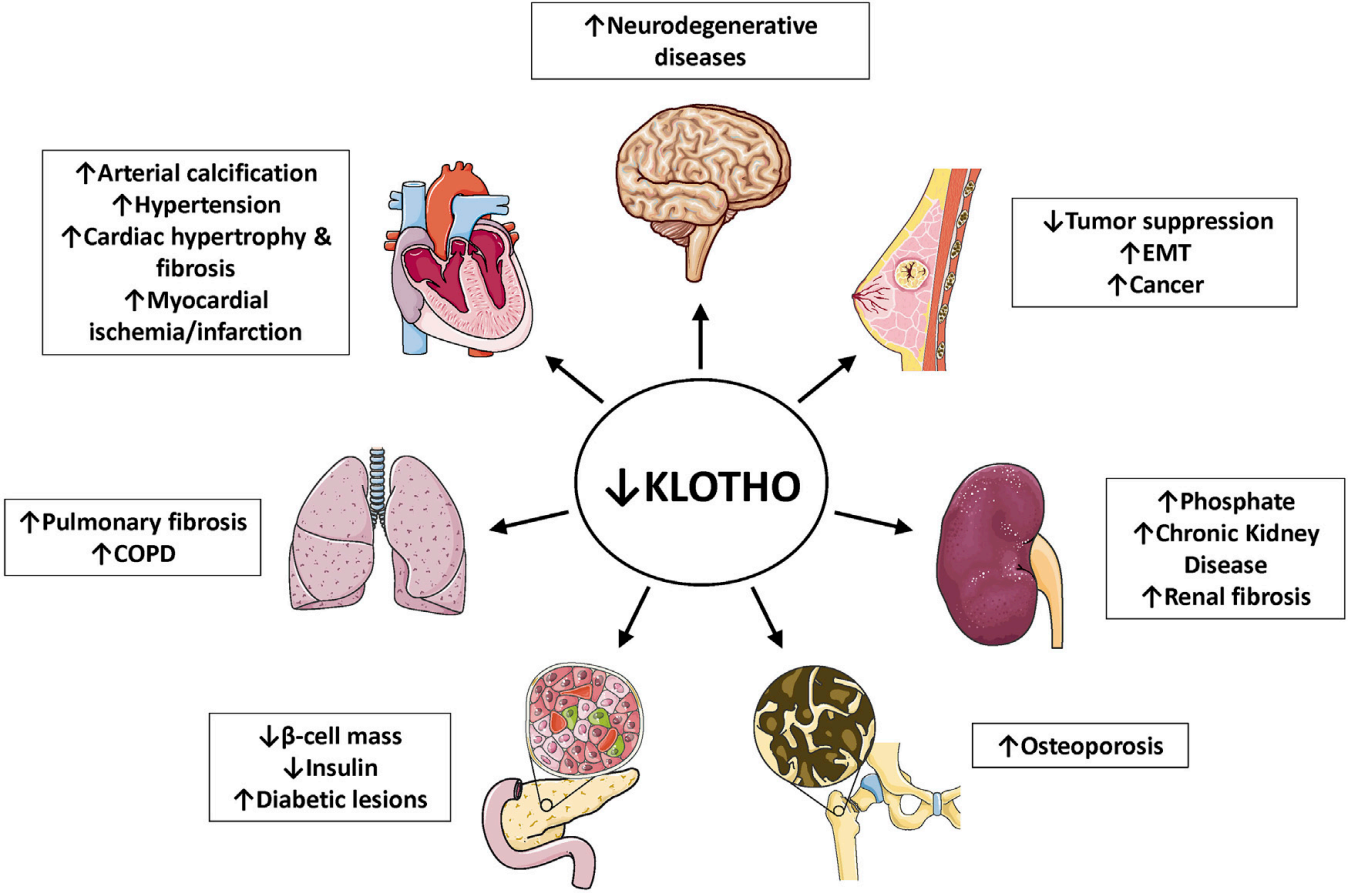
Klotho deficiency associates with multiple age-related diseases. As outlined in the captions, depressed Klotho levels are linked to hyperphosphatemia, chronic kidney diseases, multiple cardiovascular conditions, neurodegenerative diseases, several types of cancer, pulmonary fibrosis, COPD, bone disease and diabetes (reduced β-cell mass in the pancreas). References are listed in Table 1. Abbreviations: COPD, chronic obstructive pulmonary disease; EMT, epithelial-mesenchymal transition.

Klotho can exist as a membrane-bound coreceptor for fibroblast growth factor 23 (FGF23) (Urakawa et al., 2006), or a soluble endocrine mediator with many functions (Figures 1, 2) (Dalton et al., 2017; Kuro-o, 2017). Age-related deterioration of renal function results in Klotho insufficiency, and hyperphosphatemia that contributes greatly to the aging phenotype (Kuro-o 2010, 2018, 2021). Klotho protects the kidney and promotes phosphate elimination (phosphaturic effect). Remarkably, independent of FGF23, it inhibits at least four pathways that have been linked to aging in various ways. Klotho blocks or inhibits transforming growth factor β (TGF-β), insulin-like growth factor 1 (IGF-1), nuclear factor κB (NF-κB), and Wnt/β-catenin (Figure 3). Consequently, as will be presented in this review, Klotho exerts major effects on several biological processes relevant to aging and disease (Figure 4):

1) FGF23-dependent phosphate, calcium and vitamin D regulation.
2) Antioxidant and anti-inflammatory activities.
3) Prevention of chronic fibrosis.
4) Protective effects against cardiovascular disease.
5) Anti-cancer (tumor suppressor) activities.
6) Metabolic regulatory functions relevant to diabetes.
7) Anti-apoptotic and anti-senescence functions; stem cell preservation.
8) Protection against neurodegenerative disease (Alzheimer’s and other).

In addition to these topics, we will report on several factors that increase Klotho, including commonly prescribed drugs, recombinant proteins, gene therapies, traditional medicines, nutraceuticals, and exercise.

## 2 Sites of Klotho Production

Klotho is primarily produced in the kidney (renal tubules), but is also found in the brain (choroid plexus, CSF and neurons), pancreatic β cells, blood vessels and skin (Lim K, et al., 2015). Recent work also documents expression in peripheral blood circulating cells (Martin-Nunez et al., 2022). It is also excreted into the urine. Studies in nephrectomized rodents (Hu et al., 2016), and mice with kidney-specific genetic deletion of Klotho (Lindberg et al., 2014), have revealed the kidney is the principal source of circulating Klotho. It is produced by the proximal and distal convoluted renal tubules, perhaps more in the distal tubules (Lim K, et al., 2015). However, some authors report similar amounts of the membrane-bound form at both locations (Andrukhova et al., 2012; Erben and Andrukhova, 2017). Interestingly, Klotho has different functions in the proximal and distal tubules (Erben and Andrukhova, 2017). In the proximal tubule, Klotho promotes a phosphaturic effect and inhibits vitamin D production, whereas in the distal tubule it enhances Ca^2+^ reabsorption. Conditional gene knockout of *Klotho* in the proximal tubule reproduced many of the features of systemic gene knockout, confirming the importance of proximal tubular functions (Takeshita et al., 2017).

## 3 Molecular Features and Regulation

### 3.1 Membrane-Bound and Soluble Forms

Klotho consists of a single-pass membrane protein of 1,012 aa (130 kDa), in humans, with a very short intracytoplasmic segment (10 aa) (Xu and Sun 2015; Chen G, et al., 2018). The extracellular component consists of two domains, KL1 and KL2 (Figure 1). The extracellular portion can be cleaved by membrane proteases, primarily ADAM10 and ADAM17 (α-secretases), and this soluble α-Klotho form (s-Klotho) is released into body fluids where it acts as an endocrine hormone (Xu and Sun, 2015; Dalton et al., 2017). Additional cleavage might release smaller KL1 or KL2 fragments, although to our knowledge they have not been detected in the circulation. There is possibly another soluble form of Klotho; thought to be generated by alternative splicing, consisting of KL1 only and often denoted secreted Klotho. However, the sequence contains premature stop codons, and the mRNA is degraded (Mencke, et al., 2017a). Thus, it appears that the vast majority, or perhaps all, soluble Klotho consists of the KL1/KL2 form derived by proteolytic cleaving of the membrane-bound protein (shedding). Indeed, in rodents, administration of inhibitors of secretases mediating this proteolytic cleavage drastically reduce s-Klotho levels in the circulation (Hu et al., 2016).

The regulation of Klotho shedding is not fully elucidated, but the role of the α-secretase ADAM10 and ADAM17 is well documented, especially in renal tubular epithelial cells (Van Loon et al., 2015). The expression level of these enzymes might be increased in various ways, for example through the action of insulin, growth factors and cytokines (Bzowska et al., 2004; Chen CD, et al., 2007; Maretzky et al., 2008; Murphy, 2009). In contrast, tissue inhibitors of metalloproteinases (TIMPs) block the action of ADAM proteins (Chen CD, et al., 2007; Murphy, 2009). Some drugs might increase the proteolytic cleavage of Klotho. There are few published examples; however, ligustilide is a notable case. This natural compound increased the expression of ADAM10 and s-Klotho, and was protective in a mouse model of Alzheimer’s disease (see section 10.4) (Kuang et al., 2014). It is important to note that the ADAM secretase proteins have a very large number of substrates, and can modify many biological processes. Therefore, drugs targeting these enzymes are likely to lack specificity and be subject to toxicity.

The crystal structure of Klotho and its binding sites to FGF receptors (especially FGFR1c) and FGF23 have been reported (Chen G, et al., 2018). No specific receptor for s-Klotho has been identified. However, it binds to some receptors that are widely distributed, on many cell types; notably FGFRs and the TGF-β receptor.

### 3.2 Regulation of Klotho Expression

Several factors regulate Klotho expression, as reviewed (Xu and Sun, 2015). This may differ considerably in various tissues, and it is only partially understood. In the kidney, which is the main site of production, various factors can alter Klotho expression under physiological and pathological conditions, such as circulatory stress, hypertension, oxidative stress and diabetes. Klotho is generally depressed in inflammatory disorders, and is a potentially useful biomarker of inflammation (Wu and Chen, 2022).

Multiple transcription factors regulate *Klotho* gene expression, including those binding at promoter sites E-box, Ap-2, PAX4, Sp1 and Oct-1 (Xu and Sun, 2015). PAX4 is a positive regulator. There are PPAR-γ response elements in the 5′ flanking region of the gene that increase transcription. PAX4 and PPAR-γ are relevant to diabetes, as discussed in other sections. Vitamin D binds to vitamin D response elements in the promoter, to upregulate *Klotho* transcription. In contrast, NF-κB binds to the *Klotho* promoter but is inhibitory (Moreno et al., 2011; Lin et al., 2021). This might explain, at least in part, the lowered Klotho levels in inflammatory conditions. Notably, angiotensin II is a major inhibitor of *Klotho* transcription (Figure 3), and this has been attributed to the suppression of the positive regulator Sp1 (Zhou Q, et al., 2010). HMG-CoA reductase is also inhibitory and this can be reversed by statin drugs, which are inhibitors of this enzyme (Narumiya et al., 2004; Kuwahara et al., 2008) (Table 2). Statins upregulate Klotho mRNA, possibly by inactivating RhoA and/or diminishing the angiotensin II response (Narumiya et al., 2004). Interestingly, FGF23 is a strong negative regulator of Klotho transcription (Marsell et al., 2008). As a result, events that increase FGF23 production will reduce Klotho, such as chronic renal disease (Kuro-o, 2019). The *Klotho* gene is often turned off in cancer cells, possibly through a number of mechanisms (Rubinek and Wolf, 2016). In particular, the *Klotho* promoter is GC-rich and subject to DNA methylation, and in tumors it can be turned off by DNA hypermethylation. *Klotho* is also regulated by miRNAs, and this appears to be of importance in cancer (Abolghasemi et al., 2019).

## 4 Klotho Interactions With FGF23 and FGFRs

The extracellular domain of Klotho binds to either FGFR1c, -3c, or -4 (all receptor tyrosine kinases) to form a high affinity receptor for FGF23 (Figure 2) (Chen G, et al., 2018; Urakawa et al., 2006). In terms of nomenclature, FGF19, FGF21 and FGF23 are of often denoted endocrine FGFs (eFGFs), because they are soluble, enter the circulation and can act at a distance (Phan et al., 2021). FGF23 is an eFGF produced in bone that regulates renal phosphate homeostasis, together with vitamin D and parathyroid hormone (PTH) (Neyra et al., 2021; Rausch and Föller, 2022). The membrane-bound and soluble forms of Klotho can both function as coreceptors for FGF23 (Chen G, et al., 2018). Without Klotho the affinity of FGF23 for the receptor is very low. The activated FGFR signals through PI3K/Akt, phospholipase Cγ (PLCγ) and Ras/MAPK/ERK. The renal physiological functions of Klotho have been extensively reviewed (Erben and Andrukhova, 2017; Kuro-o 2017; Zou et al., 2018; Kuro-o 2019; Kuro-o 2021; Neyra et al., 2021; Saar-Kovrov et al., 2021), and are only briefly described here. Activation of FGFR1c regulates phosphate and calcium exchange in the kidney. This occurs through inhibition of the sodium-phosphate transporters NPT-2a and NPT-2c in the proximal renal tubule, thereby reducing inorganic phosphate (Pi) reabsorption (phosphaturic effect) (Erben, 2018). In the distal convoluted tubule, Ca^2+^ resorption through TRPV5 channels is enhanced. There is also in the distal tubule increased Na^+^ reabsorption, through enhanced expression of Na^+^:Cl^−^ cotransporter (NCC). Of major physiological significance, FGF23/Klotho/FGFR1c suppresses the expression of 1α-hydroxylase in the proximal renal tubule, which inhibits synthesis of active vitamin D, denoted 1,25(OH)_2_D_3_ (calcitriol). Among other effects, vitamin D promotes phosphate and calcium absorption in the gut, and this will be reduced. Furthermore, in a Klotho-independent manner, FGF23 reduces secretion of parathyroid hormone (PTH), which additionally influences phosphate calcium balance (Ben-Dov et al., 2007).

A deficiency of either Klotho or FGF23 in mice results in excessive 1α-hydroxylase activity, overproduction of active vitamin D, and associated hyperphosphatemia and hypercalcemia. In Klotho-deficient mice, hypervitaminosis D and hyperphosphatemia both appear to contribute to the accelerated aging phenotype (Kuro-o 2010, 2018, 2019, 2021). However, a low phosphate diet ameliorates disease, even though vitamin D further increases, suggesting that the hyperphosphatemia is more important than vitamin D toxicity in the aging syndrome. Nevertheless, ablation of vitamin D responses attenuated the lesions of Klotho-deficient mice (Anour et al., 2012). It has been proposed that hypersaturation of phosphate and calcium ions in the blood leads to the formation of calciprotein particles (CPPs) (Kuro-o, 2021). The CPPs precipitate in tissues, and are thought to accelerate the pathologic changes associated with aging.

The most common clinical cause of Klotho deficiency is kidney failure, which can result from many acute or chronic conditions (Neyra et al., 2021). Rare human mutations causing a severe deficiency of either Klotho or FGF23 are associated with hyperphosphatemia beginning at an early age (Ito and Fukumoto, 2021). These are autosomal recessive diseases. The subjects develop massive calcification in soft tissues, blood vessels, and in multiple organs and anatomic sites throughout the body. There are bone and dental lesions. There is also evidence of systemic inflammation. Treatment includes low phosphate diet, phosphaturic drugs, anti-inflammatory drugs, and phosphate binding agents. This severe congenital deficiency of either functional Klotho or FGF23 is not comparable to the much less reduced Klotho expression seen in chronic diseases described in this review. Indeed, these are not associated with the massive calcification observed in congenital hyperphosphatemia.

## 5 β-Klotho

The Klotho homologue denoted β-Klotho (KLB) is an obligatory coreceptor for FGF19 and FGF21, which are both eFGFs (Kilkenny and Rocheleau., 2016). KLB, unlike Klotho, does not appear to have endocrine functions on its own. FGF19 plays a major role in bile acid homeostasis and liver metabolism (Dolegowska et al., 2019; Guthrie et al., 2022). FGF21 is involved in regulating lipid metabolism, insulin secretion and glucose homeostasis (Andersen et al., 2015; Bondurant and Potthoff, 2018; Dolegowska et al., 2019). FGF21 is produced in the liver, pancreatic islets, exocrine pancreas, and other tissues. The liver appears responsible for most of the circulating FGF21. The major metabolic functions of FGF21 have received considerable attention, and it has been examined in clinical trials for the treatment of obesity and diabetes.

## 6 Klotho Functions Independent of FGF23

### 6.1 Klotho Inhibits TGF-β

A key protective function of Klotho is the prevention of fibrosis (e.g., pulmonary, renal or cardiac fibrosis), which has been attributed in large part to its ability to block TGF-β, although other pathways contribute (Huang Q, et al., 2020; Mencke et al., 2017b). TGF-β is a pleiotropic cytokine that has been linked to aging by its promotion of cellular senescence, stem cell decline, immunologic impairment, fibrosis and several other age-related pathologies (Mikuła-Pietrasik et al., 2022; Pratsinis et al., 2017; Prud’homme 2007, et al., 2017b; Tominaga and Suzuki, 2019). This is of particular interest, because Klotho binds to the type II TGF-β receptor (TβRII), which blocks TGF-β binding and receptor signaling (Doi et al., 2011) (Figure 3). In the kidneys of aging mice, Klotho levels decline, whereas TGF-β and its signaling molecules increase (Oishi et al., 2021). Administration of s-Klotho protein inhibits TGF-β signaling and protects against renal fibrosis (Doi et al., 2011). In a more specific way, a Klotho-derived peptide (30 amino acids) that inhibits TGF-β by binding to its receptor also protected against renal fibrosis (Yuan et al., 2022). These experiments provide evidence that Klotho can suppress TGF-β activities *in vivo*.

Practically all cell types express TGF-β receptors, and its importance is considerable. TGF-β signaling and its regulation are complex (Mullen and Wrana, 2017; Derynck and Budi, 2019; Aashaq et al., 2022), and only a brief outline is provided here. TGF-β is secreted in latent form, and is activated by interaction with integrins and other mechanisms. Three isoforms of TGF-β exist (TGF-β1 is the most abundant), which all bind to the same signaling receptor (Groppe et al., 2008; Huang T, et al., 2011; Aashaq et al., 2022). This receptor consists of TβRI (also denoted ALK5) and TβRII. TGF-β binds to TβRII and TβRI forming a serine/threonine kinase complex. TβRII phosphorylates TβRI, which then phosphorylates Smad2 and Smad3 (canonical pathway). Smad2 and 3 form a complex with Smad4 (the common Smad), which translocates into the nucleus. There, it binds to DNA and regulates transcriptional events. Multiple molecules interact with the TGF-β receptor complex, and can alter the binding of TGF-β, or receptor signaling, in a positive or negative fashion (Pawlak and Blobe, 2022; Prud’homme et al., 2017b). For instance, either betaglycan (also denoted TβRIII), neuropilin-1 (Nrp1) or endoglin can associate with the TGF-β receptor, acting as coreceptors, and characteristically enhance TGF-β responses. Klotho also binds to this receptor, but it has the opposite effect.

The activation of several non-canonical (non-Smad2/3 dependent) pathways adds more complexity (Derynck and Budi, 2019; Aashaq et al., 2022; Baba et al., 2022). In some cells, especially endothelial cells, signaling can occur through the alternative ALK1/Smad1,5,8 pathway. Smad4 (common Smad) then participates as it does in the Smad2/3 pathway. The signaling TGF-β receptor can activate ERK, JNK, p38 MAPK, PI3K/Akt, Rho-like GTPases, NF-κB and other pathways. Furthermore, through canonical or non-canonical signaling, TGF-β can crosstalk with bone morphogenetic proteins (BMPs), Wnt, Hedgehog, Notch, Hippo (TAZ/YAP), JAK/STAT; as well as growth factors such as hepatocyte growth factor (HGF) and epidermal growth factor (EGF) (Luo 2017; Mullen and Wrana, 2017; Derynck and Budi, 2019). Thus, TGF-β can impact on a vast number of biologic processes. In the immune system, it is produced by regulatory T cells (Treg), and other cell types, and suppresses or regulates immune responses involving macrophages, dendritic cells, B cells, effector T cells, NK cells and neutrophils (de Streel and Lucas, 2021; Maizels 2021; Prud’homme 2007). Thus, it is a key regulator of the immune system.

The studies of Klotho inhibition of TGF-β mentioned above pertain primarily to renal disease, but are likely applicable to fibrosis elsewhere such as pulmonary fibrosis, and several other age-related conditions. This would be consistent with the ubiquitous expression and multiple functions of TGF-β and its receptors. TGF-β is involved in many of the lesion observed in diabetes. The role of TGF-β in neoplasia is complex and context dependent. At early stages of neoplasia it acts as a tumor suppressor, but it aggravates cancer in the late phases, and contributes to metastatic disease (de Streel and Lucas 2021; Baba et al., 2022). Therefore, its inhibition by Klotho is relevant to cancer therapy.

### 6.2 Klotho Inhibits NF-κB

Inflammation is thought to be a major contributor to aging, in a process sometimes referred to as inflammaging (de Almeida et al., 2020; Fulop et al., 2021). Chronic, low-grade inflammation can lead to permanent tissue damage. For example, inflammatory changes of various types have been linked to atherosclerosis, chronic renal disease, diabetes-related organ injury, and Alzheimer’s disease. Klotho exerts anti-inflammatory activities that appear to be independent of FGF23. Importantly, Klotho suppresses activation of the inflammatory NF-κB pathway (Buendia et al., 2015, 2016). This pathway plays a key role in initiating immune and/or inflammatory responses mediated by B cells, T cells, macrophages and polymorphonuclear leukocytes (PMLs) (Haga and Okada, 2022; Roberti et al., 2022). It also inhibits apoptosis of immune cells and other cell types, while promoting proliferation. It is activated by T-cell and B-cell costimulatory receptors, several inflammatory cytokines, chemokines, toll-like receptors (TLR), NOD-like receptors (NLR), stimulator of interferon genes (STING), and other factors that promote immunity against infectious agents (Roberti et al., 2022; Haga and Okada, 2022; Zhang T, et al., 2021). However, NF-κB is also implicated in detrimental inflammation contributing to aging, chronic inflammatory conditions and autoimmune diseases. NF-κB is active in many non-immune cell types, such as endothelial cells and some epithelial cells. It contributes to vascular lesions (e.g., atherosclerosis and vasculitis) and, importantly, it plays a detrimental role in cancer (Zhang T, et al., 2021). Thus, NF-κB can contribute the age-related diseases in many ways.

The NF-κB family includes NF-κB1 (p50), NF-κB2 (p52), RelA (p65), and RelB, and the NF-κB activation pathways have been recently reviewed (Roberti et al., 2022; Haga and Okada, 2022; Zhang T, et al., 2021). These proteins all have a Rel homology domain for sequence-specific DNA binding, as well as homo- and hetero-dimerization. There are two routes for signaling, denoted the canonical and non-canocical (alternative) pathways. Early signaling events in the NF-κB pathways involve the activation of kinases, such as TAK1 and the NEMO complex. In the canonical pathway, in the quiescent state, the inhibitor of κB (IκB) is bound to the NF-κB (p50/RelA) and this prevents its activation. In response to stimulatory action, the IκB protein is phosphorylated, ubiquitinated, and subjected to proteasomal proteolysis. This allows NF-κB to migrate into the nucleus and bind to DNA, and induce transcription of a large number of genes. In the non-canonical pathway, RelB is bound to a protein denoted p100. The activation of NF-κB inducing kinase (NIK) leads to phosphorylation of the p100 protein, and its subsequent degradation to the p52 protein. Then, a p52/RelB complex translocates into the nucleus and activates the transcription of the target genes. Interestingly, there is evidence that the two pathways interact, and might not be completely independent.

Major inhibitory functions of Klotho derive from its ability to block NF-κB signaling, as demonstrated in several studies. In this case, Klotho appears to prevent the nuclear translocation of NF-κB, although other mechanisms may also apply, as described below. For instance, Klotho suppressed TNF-α-induced activation of NF-κB in endothelial cells (Maekawa et al., 2009). Uremia is associated with circulating toxins that cause oxidative stress to endothelial cells and induce their senescence. These endothelial changes were attributed to NF-κB activation, and this was relieved by Klotho (Buendia et al., 2015, 2016). The authors concluded that Klotho prevents nuclear translocation of NF-κB, which is an essential step in the activation pathway. Other investigators reported similar findings (Yang, et al., 2012). In a diabetic cardiomyopathy model, Klotho inhibited NF-κB *in vitro* and *in vivo* (Guo et al., 2018). This suppression appeared to be due to an increase in nuclear factor-erythroid 2-related factor 2 (Nrf2) activation, which counteracts NF-κB (see below). In diabetic db/db mice, Klotho expression was reduced in the kidney, a finding linked to increased to NF-κB activation (Zhao Y, et al., 2011). This was reversed by the application of Klotho, with a consequent reduction in inflammatory cytokines. Klotho was also found to inhibit NF-κB activation and translocation in clonal β cells (Prud’homme et al., 2017a). In that case, *Klotho* knockdown with siRNA resulted in spontaneous nuclear translocation of NF-κB p65. This is consistent with Klotho preventing the degradation of the IκB protein, as suggested by others studying alveolar macrophages (Li L, et al., 2015). Interestingly, s-Klotho added to cultures reverses the effects of *Klotho* knockdown, and prevents NF-κB activation (Li L, et al., 2015; Prud’homme et al., 2017a). The mechanisms of NF-κB inhibition in these various studies may differ, and are not fully elucidated (Typiak and Piwkowska, 2021). Furthermore, once NF-κB is activated and has translocated into the nucleus it can bind to the *Klotho* promoter and inhibit expression, as previously noted (Moreno et al., 2011; Lin et al., 2021). Thus, under strong acute or chronic inflammatory conditions NF-κB activation might be dominant and, as a result, Klotho expression depressed.

### 6.3 Klotho Activates the Nrf2 Antioxidant Pathway

Klotho activated Nrf2 in renal, cardiovascular and neurological preclinical disease models (Maltese et al., 2017; Zhu et al., 2017; Xing et al., 2021; Xiang et al., 2022). Nrf2 is a transcription factor that controls responses to oxidative stress and toxins (Dinkova-Kostova et al., 2018; Qu et al., 2020; Panda et al., 2022). Its Neh2 domains interact with a negative regulator denoted Kelch-like ECH-associated protein 1 (Keap1) (Panda et al., 2022). Keap1 binds to Nrf2, promoting its ubiquitination and proteasomal degradation. Under the influence of oxidative stress (or other activators), the thiol groups on cysteine residues in Keap1 are modified and its function is negated, resulting in the release of Nrf2. Then, Nrf2 translocates into the nucleus and forms heterodimers with other proteins, and binds to an enhancer sequence termed antioxidant response element (ARE). ARE is involved in the expression of cellular defense genes that encode, for example, several antioxidant proteins and glutathione-conjugated coenzyme There is also an alternative pathway of Nrf2 regulation not requiring Keap1. Nrf2 protects against oxidative injury and, importantly, inhibits the inflammatory NF-κB pathway (Gao W, et al., 2022). The activation Nrf2 by Klotho appears to be an important factor in the protection against renal, vascular and other diseases.

### 6.4 Inhibition of the IGF-1 Pathway

The insulin/IGF-1 receptor signaling pathway has long been linked to aging, and it is sensitive to nutrients (Mathew et al., 2017; Johnson 2018). When nutrients are abundant, the downstream mediator denoted mechanistic target of rapamycin (mTOR; a protein kinase) is activated, and it is involved in several age-related disorders. Calorie restriction (CR) can mitigate this effect, and prolong life in some species (Kapahi et al., 2017; von Frieling and Roeder, 2020). Thus, it is of considerable importance that Klotho inhibits the IGF-1/PI3K/Akt/mTOR pathway (Kurosu et al., 2005:; Xie et al., 2013). This is one likely mechanism by which Klotho exerts its antiaging action, and protection against degenerative diseases (Fung et al., 2022). In this case, s-Klotho blocks insulin/IGF-1 receptor activation, preventing downstream signaling events including phosphorylation of insulin receptor substrates (IRS) and PI3K/Akt/mTOR signaling. The mTOR inhibitory drug rapamycin delays age-related disease such as vascular calcification in mice and, interestingly, it increases Klotho expression (Zhao Y, et al., 2015). The insulin/IGF-1 pathway also plays a role in cancer progression, and its inhibition by Klotho is tumor suppressive.

The insulin/IGF-1 pathway connects with antioxidant mechanisms through the FoxO forkhead transcription factors (FOXOs). Blockade of insulin/IGF-1 pathways releases inhibition of the FOXOs, resulting in their nuclear migration and the expression of several genes encoding antioxidant enzymes, such as manganese superoxide dismutase (Yamamoto et al., 2005). In accord with this, FOXO activation is depressed in Klotho-deficient mice, but augmented in Klotho-overexpressing mice.

### 6.5 Inhibition of the Wnt Pathway

Wnt is another major pathway blocked by Klotho (Liu H, et al., 2007). Wnt is a signaling cascade involved in embryogenesis, stem cell biology, cell fate specification, polarity, mitosis and migration, as recently reviewed (Hayat et al., 2022; Rim et al., 2022). Wnt dysregulation results in anomalies of development, various degenerative conditions and cancer. There is a canonical β-catenin dependent pathway, and a non-canonical (β-catenin independent) pathway (Hayat et al., 2022). Non-canonical has two subtypes, i.e., the planar cell polarity and the Wnt/Ca^2+^ pathways. In canonical signaling, multiple Wnt ligands are able to bind to the cognate receptor, denoted Frizzled, to initiate a response. In the absence of a Wnt ligand the pathway is inactive. This is due to the constant phosphorylation of cytoplasmic β-catenin, as mediated by a protein complex, which results in its elimination by proteasomal degradation. However, following the binding of a Wnt ligand to Frizzle and coreceptor LRP, the degradation of β-catenin is terminated, and it can migrate into the nucleus to activate target genes through interactions with the T-cell factor/lymphoid enhancer-binding factor (TCF/LEF) transcription factors. This canonical pathway regulates mainly cell proliferation, involving cyclin D1, c-myc and many other proteins; whereas the non-canonical pathways regulate cell polarity and motility (Hayat et al., 2022). The proliferative aspects are particularly relevant to cancer, such as colon cancer and hepatocellular carcinoma.

Klotho blocks Wnt activation by binding to several Wnt ligands, including Wnt1, Wnt3, Wnt4, and Wnt5a (Liu H, et al., 2007; Wang and Sun, 2009). In Klotho-deficient mice, excess Wnt activation promotes cell senescence, and has a negative impact on stem cell survival (Bian et al., 2015). In the kidney, Wnt overexpression is associated with fibrosis (Li X, et al., 2021), and it collaborates with TGF-β in this process. In this respect, Wnt can also cooperate with TGF-β to induce epithelial-mesenchymal transition (EMT), which is a precursor to fibrosis (Li X, et al., 2021). Moreover, EMT has also been linked to cancer stem cell (CSC) differentiation (Prud’homme, 2012; Prud’homme, 2017b). CSCs are highly tumorigenic, promote metastasis and resist chemotherapy.

## 7 Diabetes and Obesity Relevance

Interestingly, Klotho is depleted in the islets of diabetic patients (Lin and Sun, 2015a). Several investigators have reported depressed circulating Klotho levels in subjects with type 1 diabetes (T1D) (Keles et al., 2016; Tarhani et al., 2020; Zubkiewicz-Kucharska, 2021); and type 2 diabetes (T2D) especially when there is advanced disease (Nie et al., 2017; Fountoulakis et al., 2018; Zhang and Liu, 2018). Circulating levels of s-Klotho are depressed in db/db mice (T2D) (Takenaka et al., 2011) and diabetic NOD mice (T1D) (Prud’homme et al., 2020). There is evidence that Klotho plays an important role in glucose and lipid metabolism, as reviewed (Razzaque, 2012; Donate-Correa et al., 2016; Wan Q, et al., 2017; Landry et al., 2021a). Klotho-deficient mice display islet atrophy and reduced insulin production (Razzaque, 2012). Klotho increases β-cell expression of TRPV2 and enhances Ca^2+^ entry and the glucose-induced response (Lin and Sun, 2012).

FGFR1c is expressed by β cells, and its attenuation results in diabetes and reduced numbers of β cells (Hart et al., 2000). Both Klotho and KLB are expressed by islet cells, and this suggests these cells can respond to FGF23 (Klotho coreceptor) and FGF21 (KLB coreceptor). FGFR1c promotes signaling through several key pathways for β cells (Figure 2), e.g., PI3K/Akt (promotes cell survival), RAS-MAPK (promotes mitogenic response), PLC-PPAR-adiponectin (regulates glucose and lipid metabolism). In accord with this, Klotho administered by gene transfer in mice demonstrated major protective effects on β cells in T1D or T2D models (Lin and Sun, 2015a,b). Importantly, Klotho reduced β-cell apoptosis and increased the proliferation of these cells. A key pathologic finding in NOD mice is the development of insulitis. This first appears as a peri-islet mononuclear cell infiltrate (including T cells), followed by focal invasion of immune cells into the islets, and subsequently severe infiltration and β-cell loss. Klotho gene transfer reduced insulitis, suggesting an immunosuppressive and/or anti-inflammatory effect. In addition, recombinant Klotho improved renal disease and hypertension in db/db mice (Takenaka et al., 2019). The authors concluded that Klotho inhibited TGF-β and tumor necrosis factor (TNF) signaling, to decrease renal fibrosis.

Systemic γ-aminobutyric acid (GABA) treatment protects against type 1 diabetes (T1D) in mice (Tian et al., 2004; Soltani et al., 2011; Wan Y, et al., 2015; Wang et al., 2019). It induces mouse and human β-cell replication/regeneration, while reducing the apoptosis of these cells (Liu W, et al., 2017, 2021; Purwana et al., 2014; Sarnobat et al., 2022; Soltani et al., 2011; Tian et al., 2013; Untereiner et al., 2019). In terms of mechanisms, we found that GABA increases the production of Klotho, in a multiple low-dose streptozotocin (STZ) model of T1D (Prud’homme et al., 2017a). GABA therapy increased Klotho in the pancreatic β cells, kidneys and plasma. Similarly, in the case of human islets transplanted into immunodeficient mice, we observed that oral GABA treatment increased β-cell Klotho expression and replication, and diminished apoptosis (Liu W, et al., 2021). These effects were ameliorated by co-administration of a dipeptidyl peptidase 4 (DPP-4) inhibitor.

In autoimmune diabetes-prone NOD mice, the injection of s-Klotho protein increased pancreatic β-cell replication and the β-cell mass (Prud’homme et al., 2020). It also reduced insulitis, whereas a Klotho blocking antibody had the opposite effect. These findings were similar to those of others who employed gene transfer methods (Lin and Sun, 2015a,b). *In vitro*, Klotho stimulated human β-cell survival, replication and insulin secretion (Prud’homme et al., 2017a). It also inhibited NF-κB activation, which could explain the anti-apoptotic effect. Klotho might also protect β cells through antioxidant mechanisms that can be induced, for instance, by Nrf2 and FoxO (Figure 3). Note that although GABA has only been applied in preclinical diabetes, there are numerous GABAergic drugs in clinical use (Palma et al., 2017), to treat epilepsy and other diseases. Unlike systemically applied GABA that is blocked by the BBB, many of these drugs cross this barrier and this can produce multiple adverse effects. Other than GABA, it is unknown whether GABAergic drugs increase Klotho levels.

Paradoxically, Klotho deficient mice (*KL* ^*kl/kl*^) are hypoglycemic, despite having low insulin levels (Razzaque 2012). This likely results from the fact that Klotho depresses insulin sensitivity and, therefore, the cells of Klotho-negative mice are more responsive to insulin. This raises the question of whether Klotho is an appropriate agent to treat diabetes. We hypothesize that Klotho therapy of diabetes will be helpful when normal plasma levels are restored. Interestingly, overexpression of Klotho in transgenic mice produced slight insulin resistance, and extended lifespan (Kurosu et al., 2005). In experimental T1D and T2D, as outlined in this review, Klotho therapy was beneficial. Whether humans will respond similarly is unknown. In our work, Klotho protein was administered at a low dose, every 48 h (Prud’homme et al., 2020). It has a half-life of only 7 h (Hu et al., 2016), which is relevant to efficacy and potential adverse effects.

One key issue regarding antidiabetic drugs such as GABA and GLP-1 is whether their general antidiabetic effects (Wang Q, et al., 2019; Drucker, 2018) enhance Klotho indirectly, or there is a more direct effect on Klotho gene expression. This has not been extensively studied, but we hypothesize this is due to a combination of mechanisms. In STZ-induced diabetes, STZ is toxic to the kidney (Cheng MF et al., 2010), not just pancreatic β cells, and it reduces Klotho in the circulation and kidneys (Prud’homme et al., 2017a). This was almost completely reversed by oral GABA administration. Studies of others have shown that GABA treatment protects the kidneys against tubular fibrosis and atrophy, in the subtotal nephrectomy model (Sasaki et al., 2007), and ischemia-reperfusion injury (Kobuchi et al., 2009). This is consistent with the fact that renal tubular cells express GABA receptors (Amenta et al., 1988; Sarang et al., 2008; Takano et al., 2014). Thus, GABA may be protecting renal tubular cells against injury, such that they continue producing Klotho at near-normal levels. However, GABA and/or GLP-1RA directly induced Klotho expression in pancreatic β cells. Of interest, the Klotho promoter has a transcription binding site for PAX4, which is a transcription factor essential for the differentiation of β cells in the pancreas (Sosa-Pineda et al., 1997; Xu and Sun, 2015). GLP-1 induces the expression of PAX4 in human islets (Brun et al., 2008), and therefore, this may (along with other factors) induce the expression of Klotho.

In addition to diabetes, Klotho has been found to have role in obesity. In humans, CSF Klotho levels are depressed in obese subjects (Landry et al., 2021a,b). Intracerebroventricular injection of Klotho in diabetes-prone mice reduced food intake, ameliorated glucose homeostasis and reduced body weight (Landry et al., 2020). This was related to FGFR signaling in neurons. Studies by these authors showed that Klotho acts on the arcuate nucleus of the hypothalamus (ARC) to regulate metabolism (Landry et al., 2021b). Neurons and astrocytes were both targeted, and FGFR signaling through PI3K (neurons) or ERK (astrocytes) was documented. Interestingly, they found no correlation between systemic and CSF concentrations of Klotho, and the mechanism of Klotho regulation in the CSF remains unclear. Circulating Klotho also appears to play and important role in metabolism. Systemic Klotho treatment in mice reduced adiposity and lipid accumulation in the liver; and increased lean mass and energy expenditure (Rao Z, et al., 2019). With Klotho treatment, quantitative PCR revealed lower expression of lipogenic genes.

## 8 Klotho as a Tumor Suppressor

There is considerable evidence that Klotho is a tumor suppressor molecule, as reviewed elsewhere (Rubinek and Wolf, 2016; Abolghasemi et al., 2019; Sachdeva et al., 2020; Ewendt et al., 2021). This is consistent with its capacity to inhibit pathways linked to cancer, including Wnt, IGF-1, TGF-β and NF-κB, as outlined previously (Figure 3; Table 1). The expression of Klotho is depressed or silenced in almost all types of cancer examined. Klotho-positive tumors generally have a better prognosis. The suppression of Klotho appears to occur by the same mechanism as other tumor suppressor genes. For instance, this involves DNA hypermethylation at promoter sites, histone modifications and miRNAs (Rubinek and Wolf, 2016; Abolghasemi et al., 2019). In experimental tumor models, Klotho has anti-tumor effects *in vivo* and/or *in vitro*. This was demonstrated in breast cancer (Wolf et al., 2008; Ligumsky et al., 2015), pancreatic cancer (Abramovitz et al., 2011), and other cancers including colon cancer, gastric cancer, and hepatocellular carcinoma (HCC). For instance, in pancreatic cancer, Klotho was poorly expressed in the tumor (Abramovitz et al., 2011). Overexpression of Klotho, or therapy with s-Klotho, inhibited the growth of pancreatic cancer cells. Indeed, multiple studies have shown that Klotho inhibits cancer-cell proliferation, colony formation and invasion; and promotes apoptosis and autophagy (Sachdeva et al., 2020). Similarly, *in vivo*, forced tumor overexpression of Klotho, or systemic administration of s-Klotho, inhibited the growth of several types of tumors. Importantly, this includes human tumors xenografted into mice. The membrane-bound and soluble forms of Klotho both have tumor suppressor effects.

**TABLE 1:** Klotho insufficiency and related pathologies.

Organ/system	Disease or lesion	References
Kidneys	Chronic kidney disease/fibrosis, hyperphosphatemia, ischemic injury, nephrectomy, toxic injury (adriamycin streptozotocin), diabetic nephropathy, calciprotein deposition (aging)	Cheng et al. (2010)
		Hu et al. (2013), Hu et al. (2016), Hu et al. (2017)
		Kuro-o. (2010); 2012; Kuro-o. (2019), Kuro-o. (2021)
		Maquigussa et al. (2018)
		Oishi et al. (2021)
		Prud’homme et al. 2017a, 2020
		Saar-Kovrov et al. (2021)
		Takenaka et al. (2017)
		Xing et al. (2021)
		Zou et al., (2018)
Cardiovascular	Arterial/aortic calcification, Atheroslerosis, Cardiomyopathy, Cardiac hypertrophy, Hypertension, Myocardial ischemic injury/infarct	Chen K, et al. (2021)
		Freunlich et al. (2021)
		Manrique et al. (2016)
		Myung et al. (2022)
		Martin-Nunez et al. (2022)
		Olejnik et al., 2018, 2020
		Tyrenkov et al. (2021)
		Wang F and Zheng, (2022)
		Yang et al. (2015)
Brain	Alzheimer’s disease (β-amyloid and Tau protein pathologies), hippocampal neuronal loss (Klotho promotes regeneration), cognitive deficits, frailty	Dias et al. (2021)
		Driscoll et al. (2021)
		Cararo-Lopes et al. (2017)
		Fung et al. (2022)
		Hanson et al. (2021)
		Kundu et al. (2022)
		Laszczyk et al. (2017)
		Mytych, (2022)
		Nietzel et al. (2021)
		Sanz et al. (2021)
Cancer	Loss of Klotho’s tumor suppressor function (Klotho protects against multiple cancer types)	Doi et al. (2011)
		Ewendt et al. (2021)
		Rubinek and Wolf, (2016)
		Sachdeva et al. (2020)
Lungs	Pulmonary fibrosis, chronic obstructive pulmonary disease	Huang Q. et al. (2020)
		Gao et al. (2015)
Bones	Osteoporosis, osteomalacia from chronic kidney disease	Kuro-o. (2017). 2019, 2021
		Tasnim et al. (2021)
Metabolism, Diabetes	Pancreatic β-cell apoptosis in type 1 and 2 diabetes, glucose and lipid homeostasis, regeneration of β cells, autoimmunity and inflammation (insulitis)	Keles et al. (2016)
		Landry et al., 2021a,b
		Lin and Sun, 2012, 2015a, 2015b
		Nie et al. (2017)
		Prud’homme et al. (2017a), 2020
		Razzaque. (2012); Takenata et al. (2019)

## 9 Klotho and Neurodegenerative Diseases

Low Klotho levels correlate with neurodegenerative disease and cognitive impairment (Vo et al., 2018; Hanson et al., 2021), as well as frailty (Veronesi et al., 2021). For example, in nursing home residents, low serum Klotho was associated with poor cognition, frailty, dependence, and frequent falls (Sanz et al., 2021). The role of Klotho in the brain is not well understood, but there is evidence it is neuroprotective. In the central nervous system, Klotho is produced by the choroid plexus, and is found in the CSF. It is also expressed widely throughout the brain, predominantly in grey matter areas including the hippocampus (Clinton et al., 2013; Cararo-Lopes et al., 2017). It is produced by both neurons and oligodendrocytes. Klotho expression in the brain is decreased in aging subjects and early Alzheimer’s disease (Fung et al., 2022). In mouse models of Alzheimer’s disease, Klotho overexpression is beneficial.

Morphologically, in Alzheimer’s disease, the brain is characterized by an excess of β-amyloid (Aβ) plaques, as well as neuronal aggregation of Tau protein forming neurofibrillary tangles (NFTs) (Thal et al., 2013). It has been proposed that these substances are toxic to neurons. Klotho confers neuronal resistance to oxidative and endoplasmic reticulum stress, and is thought to protect against the toxicity of Aβ and NFTs (Zeldich et al., 2014; Fung et al., 2022). Aβ is generated from the proteolytic cleavage of amyloid precursor protein (APP) by secretases (Lichtenthaler et al., 2022). APP is a type 1 transmembrane protein, and its cleavage by an α-secretase results in the release (shedding) of a large extracellular segment, which does not form amyloid. This cleavage process is accomplished primarily by ADAM10, and is similar to the generation of s-Klotho. However, processing of APP by β-secretase and γ-secretase generates small secreted Aβ peptides that form amyloid. Interestingly, α-secretase cleavage prevents the subsequent generation of amyloidogenic peptides (Lichtenthaler et al., 2022). Therefore, increasing α-secretase activity, or decreasing β- and γ-secretase activity, might be of benefit.

One potential mechanism of disease involves deficient autophagy, which is a process that normally degrades and removes proteins and other substances, preventing their toxic accumulation in the cell. Klotho ameliorates autophagy, and this might protect against neurodegenerative conditions like Alzheimer’s (Fung et al., 2022). Klotho also appears to contribute to oligodendrocytic myelin production, and the maintenance of white matter integrity. There is evidence of an inflammatory component to Alzheimer’s disease, as well as some other neurodegenerative diseases, possibly involving NF-κB activation (Ju wang et al., 2019). Thus, as in other sites, Klotho may exert beneficial anti-inflammatory and anti-oxidative actions in the brain. Klotho might promote differentiation of microglia to the anti-inflammatory type (M2), instead of the inflammatory type (M1) (Fung et al., 2022).

The hippocampus plays a major role in memory. It is one of the few regions of the brain that sustains neurogenesis in adulthood, and there is loss of neurons in that area in Alzheimer’s disease (Rao YL, et al., 2022). Remarkably, recent work indicates that Klotho stimulates neurogenesis in the hippocampus. This was evident, for example, when comparing mice with Klotho deficiency vs. those overexpressing Klotho (Laszczyk et al., 2017). In accord with neuronal loss, Klotho deficient mice demonstrate severe cognitive defects. Recently, intermittent fasting in mice was shown to increase Klotho expression and neurogenesis in the hippocampus (Dias et al., 2021). Intermittent fasting proved superior to caloric restriction, and it was associated with improvement of long-term memory. Klotho-deficient mice did not respond to intermittent fasting. It is possible that drug therapy can achieve similar results. For instance, in a rat model of metabolic syndrome, the DPP-4 inhibitor vildagliptin (increases GLP-1) augmented hippocampal Klotho, concurrently decreased inflammatory and apoptotic biomarkers at that site, and prevented neurodegenerative changes (Yossef et al., 2020). This improved memory in treated rats. Note that most DPP-4 inhibitors, including vildagliptin, do not cross the blood-brain barrier (BBB) but they enhance circulating GLP-1 (Deacon, 2019), which does cross. In a rat model of STZ-induced cognitive impairment, simvastatin increased hippocampal Klotho and improved cognitive function (Adeli et al., 2017). Similarly, others found that rosiglitazone treatment improved cerebral Klotho expression (Chen LJ, et al., 2014). In the latter study, intracerebroventricular infusion of recombinant Klotho was also beneficial. Alternatively, some authors have delivered Klotho by gene transfer (Table 2), such as intracerebroventricular injection of a lentiviral vector encoding Klotho (Zeng et al., 2019). The findings outlined above regarding Klotho in neurodegenerative pathologies have all been obtained in rodents, and it remains to be determined whether they are applicable to humans.

**TABLE 2:** Examples of current clinical drugs that increase Klotho.

Category (name)	Usual Clinical applications	References
Renin-angiotensin-aldosterone inhibitors (losartan, valsartan)	Hypertension	Janic et al. (2019)
	Chronic kidney disease	Karalliedde et al. (2013)
	Diabetic nephropathy	Li X and Zhou, (2010)
	Cardiac failure	Lim SC, et al. (2014)
		Yoon et al. (2011)
Statins (atorvastatin, pitavastatin, simvastatin fluvastatin)	Hyperlipidemia	Adeli et al. (2017)
	Atherosclerosis	Janic et al. (2019)
	Ischemic cardiovascular	Kuwahara et al. (2008)
	and renal disease	Narumiya et al. (2004)
		Yoon et al. (2012)
PPAR-y agonists (rosiglitazone, ciglitazone, pioglitazone)	Diabetes	Chen LJ, et al. (2014)
	Hyperlipidemia	Cheng et al. (2017)
		Huang KC, et al. (2014)
		Maquigussa et al. (2018)
		Shen et al. (2018)
		Zhang H, et al. (2008)
mTOR inhibitor (rapamycin, everolimus)	Immunosuppression	Mizusaki et al. (2019)
	Transplantation	Tabibzadeh, (2021)
		Tataranni et al. (2011)
		Zhao Y, et al. (2015)
Vitamin D	Vitamin D supplementation	Haussler et al. (2020)
	Hypocalcemia	Kuro-o, (2019)
	Rickets	Lerch et al. (2018)
	Osteoporosis	Tsujikawa et al. (2003)
GLP-1 receptor agonist (exendin-4) and DPP-4 inhibitors (linagliptin, sitagliptin, vildagliptin)	Type 2 diabetes	Manrique et al. (2016)
		Liu W, et al. (2017); Liu et al. (2021)
		Son et al. (2019)
		Wang Q, et al. (2019)
		Yossef et al. (2020)
Metformin	Type 2 diabetes	Xue et al. (2019)
Pentoxifylline	Peripheral vascular disease	Navarro-Gonzalez et al. (2018)
Antiplasmodial (dihydroartemisinin)	Malaria	Zhou et al. (2022)
Endothelin-1 receptor antagonist (Atrasentan)	Diabetic nephropathy	Kang and Xu, 2016

Abbreviations: DPP-4, dipeptidyl peptidase-4; GLP-1, glucagon-like peptide-1; mTOR, mechanistic target of rapamycin, PPAR-γ, peroxisome proliferator-activated receptor-γ.

To our knowledge, Klotho-based therapy has not been applied in humans with neurodegenerative diseases. Nevertheless, compared to subjects with low levels of Klotho, those with high levels (CSF or plasma) have superior cognitive functions, and a lower incidence of dementia (Semba et al., 2014; Shardell et al., 2016). It has been thought that Klotho levels in the blood and CSF are independent. Contrary to that view, some investigators have recently reported that serum and CSF levels correlate strongly, and high levels predict better cognitive function (Kundu et al., 2022). This is of major interest for future clinical studies, especially concerning drugs or other treatments that increase Klotho. A caveat is that the reasons for discrepancies between studies are not clear, concerning Klotho levels in the CSF or the circulation. There has been much concern about the specificity of some anti-Klotho antibodies used in ELISA and other assays (Kuro-o, 2019). Some authors recently examined this question in human serum samples from patients with a wide range of kidney function (Neyra et al., 2020). This was done with a widely used commercial ELISA, as well as immunoprecipitation-immunoblotting (IP-IB) with a highly specific Klotho antibody. The IP-IB Klotho results correlated with kidney function much better than the ELISA. Freeze-thaw cycles impaired Klotho detection. These findings suggest that relying solely on an ELISA assay could be misleading, and sample handling is important. Another relevant observation is the wide variability in Klotho levels between individuals. For instance, in a 18–35 age group approximately half of individuals had low levels, comparable to aged subjects, whereas the other half had high levels (Espuch-Oliver et al., 2022). In the 55–85 age group, however, levels were more consistently low. CSF levels are also quite variable (Kundu et al., 2022). In view of this, results obtained from small groups should be interpreted with caution. The biological significance of this variability is unclear, and long-term studies are required.

A *Klotho* gene variant denoted KL-VS (2 amino acid substitutions in the protein) appears to provide some protection against Alzheimer’s disease, but only in the heterozygous state (Driscoll et al., 2021; Nietzel et al., 2021). Beneficial effects were only observed in some subgroups that were studied. The mode of action of this variant is unclear although, interestingly, a reduction of Tau accumulation has been reported.

### 9.1 Summary of References for Disease Relevance

See also Table 1. Kidneys: Cheng et al., 2010; Kuro-o, 2010, 2019, 2021; Maquigussa et al., 2018; Oishi et al., 2021; Prud’homme et al., 2017a, 2020; Saar-Kovrov et al., 2021; Takenaka et al., 2017; Xing et al., 2021; Zou et al., 2018. Cardiovascular: Chen K, et al., 2021; Freunlich et al., 2021; Manrique et al., 2016; Myung et al., 2022; Martin-Nunez et al., 2022; Olejnik et al., 2018, 2020; Tyrenkov et al., 2021; Wang F and Zheng, 2022; Yang et al., 2015. Brain: Dias et al., 2021; Driscoll et al., 2021; Cararo-Lopes et al., 2017; Fung et al., 2022; Hanson et al., 2021; Kundu et al., 2022; Laszczyk et al., 2017; Mytych, 2022; Nietzel et al., 2021; Sanz et al., 2021. Cancer: Doi et al., 201; Ewendt et al., 2021; Rubinek and Wolf, 2016; Sachdeva et al., 2020. Lungs: Huang Q. et al., 2020; Gao et al., 2015. Bones: Kuro-o, 2017. 2019, 2021; Tasnim et al., 2021; Metabolism and diabetes: Keles et al., 2016; Landry et al., 2021a,b; Lin and Sun, 2012, 2015a, 2015b; Nie et al., 2017; Prud’homme et al., 2017a, 2020; Razzaque, 2012; Takenaka et al., 2019.

## 10 Approaches to Klotho-Based Therapy

### 10.1 Drugs in Clinical Use for Various Conditions

In view of the benefits of Klotho against aging and multiple diseases, as reported here in preclinical models (Figure 4; Table 1), it is of much interest of find applications in people. In humans, it is well established that plasma Klotho levels decrease with age and in common maladies, such as chronic renal diseases, diabetes and neurodegenerative diseases. In view of this, an obvious and safe goal would be to re-establish normal levels. Some drugs have already been mentioned for their potential application in specific diseases. In many of the studies reported (Table 2 and 3) the main effect was an increase in Klotho levels approaching or equal to the normal, rather than above normal. Based on studies in mice, overexpression might possibly extend lifespan, but the safety is less certain because other factors (perhaps insulin resistance) could become important. A caveat is that practically all the information available concerning Klotho-based treatment has been obtained in rodents.

**TABLE 3:** Drugs in preclinical development, supplements or other therapies that increase Klotho.

Category (name)	Target diseases	References
y-aminobutyric acid receptor agonist (GABA)	Preclinical diabetes models, streptozotocin organ injury	Liu W, et al. (2021)
		Prud’homme et al. (2017a)
		Son et al. (2019)
		Wang Q, et al. (2019)
Recombinant protein (s-Klotho, Klotho peptide, KL1)	Preclinical diabetic, metabolic, renal, vascular, neurodegenerative, neoplastic and other diseases	Ali et al. (2020)
		Chen TH, et al. (2013)
		Doi et al. (2011)
		Gupta et al. (2022)
		Hu et al. (2017)
		Leon et al. (2017)
		Maltese et al. (2017)
		Mencke et al. (2017)
		Myung et al. (2022)
		Oh et al. (2018)
		Prud’homme et al. (2020)
		Rao Z, et al. (2019)
		Takenaka et al. (2018); Takenaka et al. (2019); Takenaka et al. (2020)
		Yang et al. (2015)
		Yuan et al. (2022)
Gene therapy and cell therapy (*Klotho gene*)	Preclinical, multiple diseases (alternative to recombinant protein therapy)	Arbel Rubinstein et al. (2021)
		Franco et al. (2021)
		Lin and Sun. (2015a); Lin and Sun. (2015b)
		Ni et al. (2021)
		Mencke et al. (2017)
		Shin et al. (2019)
		Xiang et al. (2021)
		Zeng et al. (2019)
Food/diet components, supplements and traditional medicines (astaxanthin, baicalin, cordycepin, curcumin, ginseng, ligustilide, resveratrol, tetrahydroxystilbene glucoside)	Multiple indications, geroprotective	Dehghani et al. (2019)
		Feng & Huang, (2022)
		Hsu et al. (2014)
		Kamel et al. (2022)
		Long et al. (2018)
		Li SS, et al. (2018)
		Lim SW, et al. (2019)
		Long et al. (2018)
		Ostojic & Engeset, (2021)
		Zhang P, et al. (2016)
		Zhang XT, et al. (2020)
		Zhou et al. (2015)

A surprisingly large number of therapies increase Klotho levels in experimental models (Tables 2 and 3), either in the circulation or specific organs. Some of the compounds involved have received considerable attention in the literature as geroprotective molecules (Soo et al., 2020), but usually with scant examination of their Klotho-inducing capacity. This includes several drugs that are currently in clinical use for various conditions (Table 2).

#### 10.1.1 RAAS Inhibitors

RAAS inhibitors are the best documented Klotho-enhancing clinical drugs; especially losartan and valsartan that block the angiotensin II receptor (AT1). Indeed, these are some of the few drugs that have been shown to enhance plasma Klotho in clinical trials (Karalliedde et al., 2013; Lim SC et al., 2014; Janic et al., 2019). For instance, losartan increased Klotho levels by 23% in diabetic patients (Lim SC et al., 2014). This is consistent with the ability of angiotensin II to suppress the expression of Klotho (Yoon HE, 2011). Whether Klotho augmentation contributes to the clinical effectiveness of these drugs is unknown. Of note, these results were obtained in diabetic subjects, and it remains to be determined whether the drugs will act similarly in other diseases, or healthy subjects.

#### 10.1.2 Statins

Statins are another type of frequently applied drugs that increase Klotho (Table 2); however, there is limited evidence from clinical studies. Of major interest, the administration of a low-dose combination of fluvastatin and valsartan enhanced the expression of *Klotho* and *SIRT1*, which have both been linked to increased longevity (Janic et al., 2019). In contrast, *mTOR* and *NF-κB* were not increased. Statins and RAAS inhibitors are a very common drug combination in medicine, and this type of treatment might have an antiaging effect. Because these drugs have major effects on blood pressure, lipids, renal function, cardiovascular disease and other factors, it will be difficult to establish the specific contribution of Klotho.

#### 10.1.3 Pentoxifylline

Pentoxifylline has been shown to increase Klotho in a clinical trial (Navarro-Gonzalez et al., 2018). This drug is usually applied for the treatment of ischemic peripheral vascular disease, but there are other indications. In diabetic patients (type 2), serum and urinary Klotho were increased, but the change in the serum levels was relatively modest (∼6%). The patients had advanced renal disease, and this may explain the low response. In clinical trials, a major factor to consider is the health of the kidney. Renal atrophy (typically nephrosclerosis) can be severe in patients with diabetes or hypertension, and because most circulating Klotho originates from the kidney this can lower levels. Thus, low Klotho levels are likely to be a reflection of renal disease, and if the renal mass is very low then any drug treatment might fail to increase Klotho.

#### 10.1.4 Vitamin D

Vitamin D supplementation also increases Klotho, as shown in children with chronic kidney disease (Lerch et al., 2018). Children with mild to moderate kidney disease had decreased serum Klotho levels at the beginning of the study, and this was normalized by Vitamin D treatment. In contrast, those with severe renal disease had unchanged Klotho levels. This represents the limited data available from clinical work. In rodents, however, there is clear evidence of Klotho enhancement by vitamin D (Table2). To explain this therapeutic effect, the complex interactions underling FGF23/Klotho/vitamin D homeostasis must be considered, as recently reviewed (Haussler et al., 2020; Neyra et al., 2021).

#### 10.1.5 Rapamycin and Everolimus

Rapamycin (sirolimus) and everolimus are inhibitors of mTOR in clinical use, which have both been shown to increase Klotho (Table 2). Rapamycin has long been proposed as a geroprotective drug. Clinically, rapamycin is applied primarily as an immunosuppressive drug to prevent transplanted organ rejection. The treatment of renal transplant recipients with rapamycin produced hypophosphatemia and insulin resistance (Tataranni et al., 2011). Treatment of proximal renal tubular cells with rapamycin *in vitro* induced the expression of Klotho. The authors proposed that Klotho was mediating the rapamycin-related clinical alterations. In a renal transplantation clinical trial, everolimus significantly increased Klotho serum levels (Mizusaki et al., 2019). These investigators measured Klotho levels before transplantation and 1 year after. They also compared patients that were treated with everolimus versus those that were not. In both cases, Klotho levels were boosted by this drug. It is of interest that renal transplantation itself increased Klotho, in accord with nephrectomy decreasing Klotho, and other evidence that the kidney is the main source of circulating Klotho (Hu et al., 2016). The increased Klotho levels in the everolimus-treated patients did not correlate significantly with improvements in renal function, including serum creatinine, blood urea nitrogen, eGFR, calcium and phosphorus. This study was small and other immunosuppressive agents were co-administered, which limits the interpretation. In rats with chronic renal failure, oral rapamycin treatment also increased klotho, and this improved vascular calcification (Zhao et al., 2015). The benefits were attributed to Klotho and inhibition of mTOR based on a number of pharmacological and genetic methods. Notably, rapamycin could not prevent the vascular disease when Klotho expression was suppressed with siRNA, or in Klotho knockout mice. These studies point to rapamycin and everolimus as effective drugs for the enhancement of Klotho levels. These drugs, however, can have considerable adverse effects, including depressed immunity against infectious agents, and cell toxicity in some organs. For instance, rapamycin is toxic to pancreatic β cells (Prud’homme et al., 2013).

#### 10.1.6 Antidiabetic Drugs

Several clinical anti-diabetic drugs have been shown to increase Klotho expression in mice, e.g., GLP-1RAs, DPP-4 inhibitors, metformin and peroxisome proliferator-activated receptors γ (PPAR-γ) agonists (Table 2). Some were mentioned previously. Metformin, which is probably the most prescribed antidiabetic drug for T2D, increased Klotho in the circulation, kidneys and urine (Xue et al., 2019). It decreased the levels of mTOR, and this effect was reversed by Klotho suppression. Metformin has long been proposed as a geroprotective drug, although there are contradictory findings (Mohammed et al., 2021). In the case of diabetes treatment, a caveat is that metformin increases insulin sensitivity whereas Klotho has the opposite effect.

PPAR-γ is a transcription factor regulating insulin sensitivity and adipogenesis, and agonistic drugs find applications in diabetes and lipid disorders. PPAR-γ agonists are another class of drugs that enhance Klotho expression (Table 2). There is a non-canonical PPAR-responsive element in the 5′-flanking region of the human Klotho gene (Zhang H, et al., 2008), which likely explains this activity. These authors showed that *in vitro* and/or *in vivo*, troglitazone, ciglitazone and rosiglitazone increased Klotho mRNA expression. They observed increased Klotho protein in the kidneys. A PPAR-γ antagonist had the opposite effects.

##### 10.1.6.1 Summary of References for Clinical Drugs That Increase Klotho

See also Table 2. Renin-angiotensin-aldosterone inhibitors: Janic et al., 2019; Li X and Zhou, 2010; Lim SC, et al., 2014; Yoon et al., 2011. Statins: Adeli et al., 2017; Janic et al., 2019; Kuwahara et al., 2008; Narumiya et al., 2004; Yoon et al., 2012. PPAR-γ agonists: Chen LJ, et al., 2014; Cheng et al., 2017; Huang KC, et al., 2014; Maquigussa et al., 2018; Shen et al., 2018; Zhang H, et al., 2008. mTOR inhibitors: Mizusaki et al., 2019; Tabibzadeh, 2021; Tataranni et al., 2011; Zhao Y, et al., 2015. Vitamin D: Haussler et al., 2020; Kuro-o, 2019; Lerch et al., 2018; Tsujikawa al., 2003. Antidiabetic drugs: Manrique et al., 2016; Liu W, et al., 2017, 2021; Son et al., 2019; Wang Q, et al., 2019; Yossef et al., 2020; Xue et al., 2019. Pentoxifylline: Navarro-Gonzalez et al., 2018. Other: Zhou et al., 2022; Kang and Xu, 2016.

### 10.2 Drugs in Preclinical Development

Numerous drugs, recombinant proteins (or peptides) and gene therapies have been tested in preclinical disease models for their ability to boost Klotho levels (Table 3). Specific examples of the administration of recombinant s-Klotho or peptides were discussed before. Gene therapy has been performed with viral vectors or plasmids, and cell therapy also appears feasible (Franco et al., 2021). In some cases, such as recombinant protein and gene therapies, the feasibility, potential adverse effects, accessibility, and high cost are likely to be limiting factors.

Recombinant Klotho protein therapy has been successfully applied to the treatment of renal, cardiovascular and neurodegenerative disease, as well as diabetes and cancer (Table 3). Generally, these have been short-term study, with injection of s-Klotho, the KL1 domain, or relevant peptides. Low doses are usually effective. Recombinant protein has been delivered systemically, or in the ventricles of the brain for neurological diseases.

Common food/diet components (e.g., astaxanthin, curcumin and resveratrol), and traditional medicines and/or their extracted compounds (e.g., baicalin, cordycepin, ginseng and ligustilide), have been examined and successfully induced Klotho (Table 3). Some of these are frequently incorporated into over-the-counter nutraceuticals. In general, these compounds ameliorated or restored Klotho levels in rodent disease models where they are depressed. This work was performed in animal models, and it is largely unknown whether these substances can increase Klotho in humans.

### 10.3 Exercise and Fitness

Of major interest, physical exercise and sports are simple ways to increase circulating Klotho, as reviewed by others (Amaro-Gahete et al., 2018). A single bout of exercise transiently increases circulating Klotho (Tan et al., 2018; Morishima and Ochi, 2021). The FIT-AGEING study examined the association between physical activity and fitness and plasma levels of s-Klotho in middle-aged sedentary adult people (Amaro-Gahete et al., 2019a,b). The authors reported that higher activity and fitness associated with increased s-Klotho levels. They assessed the effects of different courses of exercise over a 12-week period, and found that all types increased Klotho, as compared to no exercise. They report that higher s-Klotho associates with decreased fat mass and increased lean mass.

#### 10.3.1 Summary of References for Drugs or Treatments in Development

See also Table 3. GABA receptor agonist: Liu W, et al., 2021; Prud’homme et al., 2017a; Son et al., 2019; Wang Q, et al., 2019. Recombinant Klotho protein or peptides: Ali et al., 2020; Chen TH, et al., 2013; Doi et al., 2011; Gupta et al., 2022; Hu et al., 2017; Leon et al., 2017; Maltese et al., 2017; Mencke et al., 2017; Myung et al., 2022; Oh et al., 2018; Prud’homme et al., 2020; Rao Z, et al., 2019; Takenaka et al., 2018, 2019, 2020; Yang et al., 2015; Yuan et al., 2022; Zhong et al., 2020. Gene therapy: Arbel Rubinstein et al., 2021; Franco et al., 2021; Lin & Sun, 2015a, b; Ni et al., 2021; Mencke et al., 2017; Shin et al., 2019; Xiang et al., 2021; Zeng et al., 2019. Traditional medicines, nutraceuticals and supplements: Dehghani et al., 2019; Feng and Huang, 2022; Hsu et al., 2014; Kamel et al., 2022; Long et al., 2018; Li SS, et al., 2018; Lim SW, et al., 2019; Long et al., 2018; Ostojic and Engeset, 2021; Zhang P, et al., 2016; Zhang XT, et al., 2020; Zhou et al., 2015. Exercise and sports: Amaro-Gahete et al., 2018, 2019a,b; Morishima and Ochi, 2021; Tan et al., 2018.

### 10.4 The BBB and Treatment of Neurodegenerative Diseases

The BBB (blood-brain barrier) blocks entry of the majority of drugs into the brain, and represents a challenge for the therapy of neurodegenerative conditions, as reviewed (Partridge, 2012; Nilles et al., 2022). Some small lipid-soluble drugs cross by lipid-mediated diffusion.

Other drugs may be shuttled by specific transporters. Large drugs (usually proteins) generally do not cross, or transfer very inefficiently, but transfer can be improved by engineering constructs for receptor-mediated transport. In addition, neuropilin-1 appears to facilitate entry into the brain of some cell-penetrating peptides, larger molecules, or viruses (Balistreri et al., 2021; Prud’homme and Glinka, 2012; Ruoslahti, 2017; Zhao L, et al., 2021).

RAAS blocking drugs represent an interesting example, because they can be divided into BBB-crossing and non-crossing types. For example, the AT1-receptor blockers losartan, irbesartan, olemesartan and eprosartan do not penetrate through the BBB, whereas valsartan, telmisartan and candesartan do cross (Ho et al., 2017). Memory is improved in older adults treated with AT1 blockers, and users of the BBB-crossing drugs performed better than those taking non-crossing drugs (Ho et al., 2017). The subjects receiving BBB-crossing drugs also had significantly fewer white matter hyperintensities (WMH) upon imaging. Other investigators (Ohrui et al., 2004) reported similar findings in patients receiving BBB-crossing angiotensin-converting enzyme (ACE) inhibitors. Those treated with the BBB-crossing drugs (captopril, perindopril) had a significantly lower risk of developing Alzheimer’s disease, compared to those taking non-crossing drugs (imidapril, enalapril). The protective mechanism is unclear. Can the BBB-crossing RAAS inhibitory drugs increase Klotho in the brain or CSF? To our knowledge, there is no data in humans. In the cerebrum of spontaneously hypertensive rats, however, valsartan treatment reduced cell damage, as determined by ultrastructural changes such as apoptotic body formation (Li and Zhou, 2010). Valsartan treatment was associated with increased intra-cerebral expression of Klotho, as determined by RT-PCR, immunohistochemistry, and Western blotting. This provides some evidence, albeit limited, that valsartan can increase Klotho expression in the brain. Statins are thought to have at least some neuroprotective effects. They can also be divided in BBB-crossing and non-crossing. The lipophylic statins (e.g., atorvastatin, lovastatin, simvastatin) cross, and the hydrophilic ones do not (Wood et al., 2010). In this case, simvastatin increased Klotho in the hippocampus of rats (Adeli et al., 2017). It is thus conceivable, but not yet certain, that both RAAS inhibitors and statins can enhance Klotho expression in the human brain, and this might protect against neurodegenerative diseases.

Some antidiabetic drugs (or at least members of these drug families) that enhance circulating Klotho levels in rodents can cross the BBB. This includes some GLP-1RAs (Pujadas and Drucker, 2016), metformin (Moreira, 2014), and at least one DPP-4 inhibitor (omariglipin) (Ayoub et al., 2018). DPP-4 inhibitors increase GLP-1 (Deacon, 2019), the natural GLP-1RA, which crosses the BBB. These antidiabetic drugs possibly ameliorate neurodegenerative disease in some patients (Mehan et al., 2022), but a potential contribution of Klotho, to our knowledge, has not been examined.

As shown in Table 3, several compounds derived from traditional medicines, or other sources, have been shown to increase Klotho expression in rodents. Interestingly, some of these compounds have been reported to cross the BBB, such as astaxanthin, baicalin, cordycepin, ligustilide and resveratrol. Curcumin (Reddy et al., 2018) as well as a component of ginseng, ginsenoside RG1 (Zhao YN, et al., 2018), also appear to cross the BBB at least to some extent. Ligustilide is of special interest, because it readily crossed the BBB and was protective in mice against ischemic brain injury and Alzheimer-like disease. In the ischemic brain, NF-κB and inflammatory changes were reduced (Long et al., 2018). In the Alzheimer’s disease model, IGF-1/Akt/mTOR signaling was inhibited (Kuang et al., 2014). Ligustilide increased Klotho in the choroid plexus. Downregulation of Klotho reduced the protective effects. The neuroprotection appears to depend at least in part on an enhancement of ADAM10 activity. As noted previously, α-secretase cutting of APP by ADAM10 produces shedding of a large non-pathogenic segment, and prevents the generation of small Aβ-forming peptides (Lichtenthaler et al., 2022). Ligustilide treatment enhanced ADAM10 activity resulting in the higher production of both s-Klotho and non-amiloidogenic APP. Aβ plaque formation was reduced in the ligustilide-treated mice. The authors (Kuang et al., 2014) concluded the enhancement of s-Klotho and α-processing of APP both contributed to the amelioration of the neurodegenerative disease.

In some cases, drugs that do not cross the BBB can ameliorate Alzheimer’s disease, or other neurodegenerative conditions, by indirect mechanisms. The improvement of hypertension, hyperlipidemia, diabetes or renal function can all have a major impact on the brain. In young and aged mice, systemic delivery of a Klotho-derived peptide that does not cross the BBB improved cognition and reduced neurodegenerative pathology (Leon et al., 2017). Others showed that systemic delivery of either s-Klotho or only the KL1 domain also improved cognition (Gupta et al., 2022). It is unclear how these beneficial Klotho-based effects are mediated, but penetrance into the brain does not appear to be required, and this merits further investigation.

## 11 Conclusion and Future Directions

In this manuscript we explored the many facets of Klotho biology. The best characterized aspects relate to renal physiology, and especially phosphate and calcium homeostasis. The importance of the FGF23/Klotho/FGFR interaction was evident in Klotho deficient mice. The major outcomes were hyperphosphatemia and hypervitaminosis D. The accelerated aging syndrome of these mice was greatly ameliorated by a low phosphate diet. Rare mutations of either *Klotho* or *FGF23* in humans result in a hyperphosphatemic syndrome, characterized by massive tissue calcification and systemic inflammation. In chronic renal disease of various etiology, as well as diabetes, Klotho production is decreased. It has been proposed that declining renal function, and associated low Klotho, accelerate or aggravate many of the pathologies found in old age. This may involve, for example, the precipitation of CPPs in tissue (Kuro-o, 2021).

It seems unlikely that all the manifestations of Klotho insufficiency are related to FGF23-induced signaling. Indeed, as outlined in several sections, Klotho blocks major pathways that play a role in aging. This refers specifically to inhibition of TGF-β, Wnt, IGF-1 and NF-κB. These are complex pathways that impact on a vast number of biological processes. Thus, regardless of other mechanisms that may drive aging, the overactivation of these pathways is likely to be important. This is most evident in fibrosis, inflammation and cancer, which are all countered by Klotho. Furthermore, Klotho activates antioxidant pathways, such as Nrf2 and FOXOs, which are thought to mediate antiaging effects.

In terms of future directions, there are several important avenues. Klotho is described as an antiaging molecule, although there is no direct evidence that it delays aging in humans. This is one of the most important questions to address, but it will require very long-term studies. Klotho can be a marker of disease (kidney, brain or other), or a therapeutic molecule. In particular, the function(s) of Klotho in the brain are not well defined, but neuroprotective effects are apparent. Klotho levels in the circulation or CSF could indicate susceptibility to disease. Multiple drugs appear to increase Klotho, but studies in humans are scant. Does Klotho contribute to the therapeutic effects of some drugs used to treat hypertension, hyperlipidemia, diabetes or transplant rejection? Could some of these drugs ameliorate neurodegenerative diseases by enhancing Klotho? This is all unknown. Indeed, there have been practically no clinical investigations of Klotho-related treatment, outside of preclinical work. Another important aspect is the role of Klotho as a tumor suppressor molecule. This is well documented in animals models, but needs to be developed in the clinical context. The slim clinical applications of Klotho research can perhaps be explained by the limited understanding of its mechanisms of action, especially outside the kidney. However, knowledge of the molecular and physiological aspects of Klotho has progressed considerably in recent years, as outlined in this review.

For therapy, several approaches are feasible, as described in this manuscript. Clinical research focused on any of these approaches would be an important contribution to the field. The most direct approach is the administration of recombinant Klotho or peptides. In view of the relatively short half-life of these products, there is a need to produce long-acting constructs, or slow-release formulations. There is little evidence from preclinical work that Klotho administration is toxic, at least when restoring normal levels. However, the long-term effects of supra-normal levels are not known. Gene therapy is also feasible, but much more challenging. In addition to some clinical prescription drugs, there are also commonly available over-the-counter medicines, supplements, or nutraceuticals that increase Klotho. This is based on animal work, and it would be of much interest to examine the possibility of Klotho enhancement in humans. In the case of neurodegenerative diseases it is relevant that some drugs, including some traditional medicines, cross the BBB and might increase Klotho in the brain. Finally, exercise and the long-term maintenance of fitness are simple methods to increase Klotho. In conclusion, most of the studies on Klotho have been performed in animal disease models, and a considerable amount of work has to be performed to introduce Klotho therapy into the clinic. This review provides a framework of mechanisms and findings that favor the clinical investigation of Klotho-based treatments.
